# Evidence-based interventions in primary care following acute coronary syndrome in Australia and New Zealand: a systematic scoping review

**DOI:** 10.1186/s12872-016-0388-y

**Published:** 2016-11-09

**Authors:** Manavi M. Bhagwat, John A. Woods, Mithilesh Dronavalli, Sandra J. Hamilton, Sandra C. Thompson

**Affiliations:** 1 Western Australian Centre for Rural Health, The University of Western Australia (M706), 35 Stirling Highway, Crawley, Western Australia 6009 Australia; 2 Georgetown University, Washington, DC USA

**Keywords:** Acute coronary syndrome, Australia, Cardiovascular disease, Evidence-based medicine, Ischaemic heart disease, Myocardial infarction, New Zealand, Primary care, Secondary prevention

## Abstract

**Background:**

Coronary artery disease has a significant disease burden, but there are many known barriers to management of acute coronary syndrome (ACS). General practitioners (GPs) bear considerable responsibility for post-discharge management of ACS in Australia and New Zealand (NZ), but knowledge about the extent and efficacy of such management is limited. This systematic review summarises published evidence from Australia and New Zealand regarding management in primary care after discharge following ACS.

**Methods:**

A search of PubMed, Scopus, CINAHL-Plus and PSYCINFO databases in August 2015 was supplemented by citation screening and hand-searching. Literature was selected based on specified criteria, and assessed for quality using the Mixed Methods Appraisal Tool (MMAT). Extracted data was related to evidence-based interventions specified by published guidelines.

**Results:**

The search yielded 19 publications, most of which reported on quantitative and observational studies from Australia. The majority of studies scored at least 75 % on the MMAT. Diverse aspects of management by GPs are presented according to categories of evidence-based guidelines. Data suggests that GPs are more likely to prescribe ACS medications than to assist in lifestyle or psychological management. GP referral to cardiac rehabilitation varied, and one study showed an improvement in the number of ACS patients with documented ACS management plans. Few studies described successful interventions to improve GP management, though some quality improvement efforts through education and integration of care with hospitals were beneficial. Limited data was published about interventions effective in rural, minority, and Indigenous populations.

**Conclusions:**

Research reflects room for improvement in GP post-discharge ACS management, but little is known about effective methods for improvement. Additional research, both observational and interventional, would assist GPs in improving the quality of post-discharge ACS care.

**Electronic supplementary material:**

The online version of this article (doi:10.1186/s12872-016-0388-y) contains supplementary material, which is available to authorized users.

## Background

Modern medical treatments like coronary revascularization for acute coronary events have benefits of high rates of patient recovery but there are high risks of hospital readmission and mortality for survivors. For example, a recent follow up of patients undergoing percutaneous coronary interventions found that 4.7 % were readmitted within 30 days, and nearly half of these (2.1 %) were classified as ACS/heart failure related [1]. For this reason, it is imperative that patients receive proper medical management of coronary risk factors and support for the adoption of a healthy lifestyle [2–4].

Based upon good evidence, guidelines recommend comprehensive post-discharge ACS care that covers management of biomedical and lifestyle risk factors, pharmacotherapy, psychological factor assessment, and assistance in initiating and maintaining behaviour change [5, 6] (Table 1). Interventions recommended by the guidelines are known to reduce patients’ risk of subsequent cardiac events [5, 7–9]. However, since hospital stays for ACS are decreasing in length, much of the responsibility for post-discharge management is left to the general practitioner (GP).

**Table 1:** Evidence-based interventions for acute coronary syndrome in primary care [5, 6]

Category	Specific Areas for GP Actions
Lifestyle/Behavioural Risk Factors/Medical Management	Smoking Cessation
	Nutrition Advice
	Alcohol
	Physical activity
	Weight Management
Pharmacotherapy	Lipid management
	Blood pressure management
	Diabetes management
	Antiplatelet agent prescription
	ACEi/ARA prescription
	Beta-blocker prescription
	Statin prescription
	Short-acting nitrate prescription
Psychological Management	Depression management
	Social Support
Behaviour Change	Referral to cardiac rehabilitation
	Chest pain action plan

*ACEi/ARB* angiotensin-converting enzyme inhibitor/angiotensin-II receptor blocker

The implementation of recommended interventions is imperfect [10, 11] as there are many barriers and facilitators to post-discharge management of CVD. ACS is usually treated in-hospital, and so primary care management depends on the receipt of informative discharge summaries from medical specialists. Additionally, comorbidities like diabetes and depression often make ACS management in primary care more complicated [12].

Adherence to these evidence-based guidelines has been shown to vary in different populations [13]. Australia and New Zealand’s populations enjoy comparable health status and both have universal public health coverage [14, 15]. In both these nations, coronary artery disease is a top health system priority. In Australia, coronary heart disease is responsible for over 10,000 deaths every year, and this number is expected to reach 13,675 by the year 2020 [16]. However, several factors complicate ACS management in these nations, with both Australia and New Zealand’s health systems having large rural populations. This may require patients to travel considerable distances to access health services like cardiac rehabilitation (CR), and there are known challenges around integration of hospital and primary care management in rural areas. In addition, both nations have significant Indigenous populations that carry a disproportionate burden of CVD [17, 18] with poorer socioeconomic circumstances compared to non-Indigenous people [19]. Both Australia and New Zealand’s health systems have funding complexity for health services [14, 15] despite significant government funding to support access to healthcare for citizens.

Studies that attempt to document ACS management often focus on particular aspects like drug utilization [20, 21] and CR referral and attendance [22]. However, little is known about overall general practice management of ACS, especially in the context of the health systems of Australia and New Zealand. Syntheses of primary care research have been shown to be useful in shaping health policy initiatives [23]. This study aims to synthesize, using a systematic approach, knowledge about evidence-based post-discharge treatment of ACS in primary care settings in Australia and New Zealand.

## Methods

### Information sources and search strategy

A systematic literature search was conducted using the following electronic databases: PubMed, SCOPUS, PsychINFO and CINAHL. The search terms comprised subject headings specific to databases where applicable, such as Medical Subject Headings (MeSH) in PubMed, as well as synonyms for these terms generated by the authors or listed in the databases. These searches were supplemented by citation screening of retrieved records and additional hand searching.

Records retrieved were those containing search terms related to ACS, patient discharge/post-discharge management, and either primary care, secondary prevention, and/or cardiac rehabilitation (CR). CR was included as a domain in the search strings because, although generally defined as medically supervised programs, CR services are sometimes expanded to include many different aspects of post-discharge management of ACS [24]. The literature search was limited to studies conducted in Australia or New Zealand and to journal articles published in the English language from the year 2000 onwards.

The literature search was last conducted on August 18^th^, 2015. An example of a search string is presented in Additional file 1.

### Study selection and inclusion criteria

**Fig. 1:**
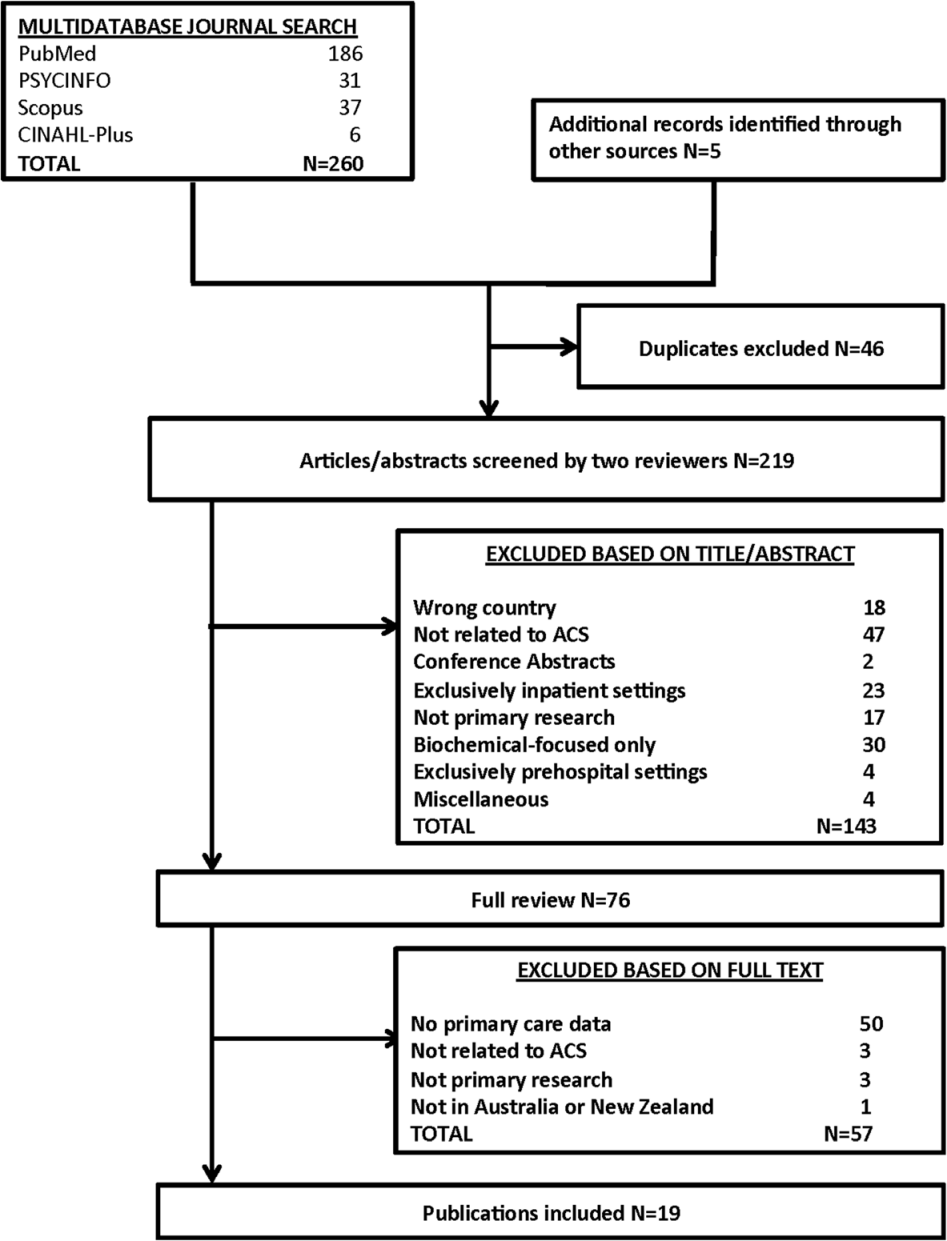
Flowchart of Search Strategy and Output: PRISMA flowchart

Duplicates were identified and removed. The remaining titles and abstracts were screened for eligibility and those that did not meet the inclusion criteria (Table 2) were excluded. Two reviewers (JW and MB) conducted this initial screening process independently, with any discrepancies resolved by discussion. Full-texts of the remaining publications were retrieved and assessed by three authors (MB, MD, JW) against the inclusion and exclusion criteria. A flow diagram of the literature search and selection process is presented in Fig. 1.

**Table 2:** Literature screening (PICO) criteria

	Inclusion	Exclusion
Population (P)	-ACS patients -GPs treating patients post-ACS -Studies conducted in Australia or New Zealand -Any age, race, sex	-Other cardiac conditions -Heart failure -Atrial fibrillation -Non-acute coronary artery disease -Other acute illnesses
Intervention (I)	Care/Interventions [5]: -Pharmacotherapy -Lifestyle management -Behaviour change -Psychological assessment	-Aspects of evidence-based management not undertaken in a primary care setting
Comparator (C)	(not applicable)	(not applicable)
Outcome (O)	-Clinical indicators of care, including: -Medication prescription rates including LLT -Smoking cessation/advice rates - Lifestyle advice receipt -CR referral rates -Depression, anxiety, stress rates -Rates of psychological assessment -Dietary advice rates -Insights about barriers and facilitators to primary care management	-Comparisons of efficacy of pharmaceuticals -Indicators measured in hospital or at discharge -Indicators in pre-hospital care -CR attendance with no primary care intervention
Study Design	-Cohort -Cross sectional -Quality improvement -Quantitative or qualitative studies	-Conference abstracts -Systematic reviews -Case reports

*ACS* acute coronary syndrome, *GP* general practitioner, *LLT* lipid-lowering therapy, *CR* cardiac rehabilitation

### Data extraction and appraisal

Data extraction was undertaken by all authors utilising a template with studies grouped according to the type of evidence-based primary care intervention they described. Studies were assessed for quality by one author (MB) based on the Mixed Methods Appraisal Tool (MMAT) [25], a method of appraising studies of various designs, in consultation with a second author (JW).

## Results

Of a total of 219 publications identified, 76 underwent full review and 19 publications reporting on 17 studies met the inclusion criteria (Fig. 1). Most studies were Australian (*n* = 15), quantitative (*n* = 16), and observational (*n* = 16). Only one randomized controlled trial was identified and there was only one qualitative study. Few studies examined Indigenous (*n* = 3), other non-Indigenous minority, or rural (*n* = 5) populations. Two studies examined exclusively female populations, and most studies were constructed with patients (*n* = 15) rather than GPs (*n* = 4) as study populations. A description of studies is presented in Table 3. Studies that reported including male and female populations consistently included a majority of male participants (*n* = 12).

**Table 3:** Included studies of primary care post-discharge management of acute coronary syndrome

First Author (Year)	Study Design	Location	Participants	Evidence-Based Intervention(s) (per NHF/CSANZ Guidelines [5])
Cole (2014) [26]	Cohort	Melbourne, Australia	12,813 PCI patients in the Melbourne Intervention Group registry	Pharmacological management
Fernandez (2006) [38]	Cross-Sectional: consecutive case series	Sydney, Australia	202/275 PCI patients who agreed to participate	Lifestyle management Psychological management
Ford (2011) [27]	Cohort with 3 year follow up	Auckland, NZ	112 ACS patients	Lifestyle management Pharmacological management
Gallagher (2003) [35]	Cohort; Mixed Methods	Sydney, Australia	196 female CR participants	Behaviour change
Hansen (2011) [43]	Qualitative	Tasmania, Australia	35 ACS patients who were smokers at time of hospitalisation	Lifestyle management
Hickey (2004) [30]	Cross-Sectional: indicators of care	Brisbane, Australia	104 ACS patients	Lifestyle management Pharmacological management
Johnson (2010) [42]	Retrospective analysis of registry data combined with (self-report) survey	Hunter, Australia	4971 patients eligible for CR	Lifestyle management
Looi (2011) [28]	Retrospective cohort, data from hospital CCU database	Auckland, NZ	129 of 901 patients with ACS who received inpatient CABG	Pharmacological management
Mudge (2001) [33]	Retrospective cohort	Brisbane, Australia	282 of 352 ACS patients with follow-up information available	Pharmacological management Biomedical risk factor management
Reddy (2008) [39] (further details in [48])	Descriptive short report of an intervention using mixed methods	Victoria and South Australia	36 health professionals	Psychological management
Rushford (2007) [36]	Cross-Sectional; Mixed Methods	Melbourne, Australia	224 female ACS patients	Behaviour change
Schrader (2005) [41]	RCT	Adelaide, Australia	669 cardiac patients	Psychological management
Schulz (2000) [37]	Cross-Sectional: follow-up survey	Horsham, Australia	79 MI patients	Behaviour change
Scott (2004) [31]	Before-after evaluation of a quality improvement program of in-hospital & post-discharge care for cardiac patients (ACS or HF)	Brisbane, Australia	344 ACS patients (of 662 eligible) who had evaluable follow-up data	Behaviour change Pharmacological management
Toms (2003) [34]	Cross-Sectional	Canberra, Australia	93 MI patients	Behaviour change Pharmacological management Lifestyle management
a. Wachtel (2008) [29] b. Wachtel (2008) [40]	Retrospective cohort: analyses of hospital records & follow-up in GP clinics	Riverland, Australia	34 MI patients with GP records, of 55 with hospital records, of 77 eligible participants	Lifestyle management Psychological management
a. Peterson (2012) [32] b. Wai (2012) [20]	Before-after evaluation of a quality improvement program discharge care (ACS)	Australia	Pre: 49 hospitals; 1545 ACS patients recruited Post: 45 hospitals remained in program; 1589 ACS patients recruited	Behaviour change Pharmacological management

*ACS* acute coronary syndrome, *CABG* coronary artery bypass grafting, *CCU* coronary care unit, *CR* cardiac rehabilitation, *HF* heart failure, *MI* myocardial infarction, *NZ* New Zealand, *PCI* percutaneous coronary intervention, *RCT* randomised clinical trial

Many publications reported studies on efforts to improve patient follow-up care that were directed from hospitals where patients with ACS had been treated, and these contained limited information regarding specific primary care involvement. Findings are reported in Table 4 and below, categorised according to areas of evidence-based management specified by the Cardiac Society of Australia and New Zealand (CSANZ) guidelines [2].

**Table 4:** Key findings on primary care post-discharge management of acute coronary syndrome

First Author (Year) Aim or Research question	Key findings on ACS interventions in primary care	Principal conclusions	Study Quality Comments MMAT Score
Cole (2014) [26]	Significant increase (*p* < 0.01) in frequency of use of all EBM investigated during calendar period 2005–2010 Medication usage at 12 months post-ACS (2010): Aspirin 96 % Clopidogrel 71 % DAPT 68 % βB 70 % ACEi/ARB 80 % Statin 93 %	Guideline-indicated medication use has increased over the 6-year study period, but treatment gap remains	• Data extracted from pre-existing ACS follow-up registry • 89 % follow-up MMAT: 100 %
Fernandez (2006) [38] To investigate risk factor status of post-PCI patients	Risk factor status at 1 year post-PCI: Systolic blood pressure above target 31 % Total cholesterol above target 58 % Smoking 15 % BMI above healthy range 77 % Obesity 34 % Physical activity below target 48 % Depression & anxiety 25 % One third of patients erroneously believed that they had no heart problems	There is inadequate management of identifiable risk factors among post-PCI patients 12–18 months after revascularisation	• 39 % response among eligible participants • Self-reported risk factor status in self-administered questionnaire MMAT: 75 %
Ford (2011) [27] To measure attainment of New Zealand Guideline Group targets & highlight areas of weakness	Risk factor status at 3 years post-ACS (2010): Attainment of target blood pressure 76 % Smokers who quit 52 % BMI in target range 24 % HDL levels above target 74 % LDL levels below target 52 % In 2010, at 3 years post-ACS, % of medications not prescribed by GPs: Aspirin 1 % βB 6 % ACEi/ARB 22 % Statin 3 % GTN spray 27 %	Concern that GPs were using outdated guidelines Mixed achievement of NZGG program—large treatment gaps for BMI, HbA1c & lifestyle Need for further efforts to improve diet & exercise Weight reduction particularly challenging—majority of patients remained overweight/obese	Reports data by ethnicity Survivorship bias (26 patients had died) Interventions implied to be based in primary care MMAT: 75 %
Gallagher (2003) [35] To identify determinants of women’s attendance at CR and adherence to risk factor modification	At 12 weeks post-discharge: • Two-thirds of women referred to CR • Only one third of the total sample attended CR • CABG patients more likely to be referred than MI patients • Lack of employment, age <55 or >70 and stressful personal life event decreased the odds of attending	Good adherence to guidelines on medications, stress modification & smoking Poorer adherence to diet & exercise guidelines	Self-reported outcomes Non-English speakers excluded MMAT: 100 %
Hansen (2011) [43] To investigate experiences of ongoing smoking or smoking cessation post-ACS	In 2006–2008, insights about GP smoking advice to patients post-ACS: • GP advice sometimes resented and sometimes appreciated • GPs more likely to talk to than lecture at patients compared to specialists • Doctor patient rapport is important • Majority of quitters spontaneously quit with no GP advice • Failed quitting attempts lead to hopelessness	Being bombarded with anti-smoking advice during hospitalisation can result in patients “turning off” Anti-smoking advice may have a positive cumulative effect when presented well & at the right time Pharmacotherapy is underutilised GPs could better inform patients about the process of quitting & available supports	• Appropriate subject selection • Low dropout • No comments on how researchers could influence interview responses MMAT: 75 %
Hickey (2004) [30] To determine whether reliable and valid clinical indicators could measure ACS primary and hospital care To determine whether education efforts could improve these clinical indicators	In 2002, insights from a program for hospitals and GPs: • Robust process and outcome clinical indicators can be developed to assess primary and hospital care that are relevant, reliable, valid and high impact • Education program improved 17/40 developed indicators	Suboptimal performance was improved with feedback to GPs. Economical data collection and timely feedback would improve QI process Sustainability of this approach limited by expense and labour	• Listed strategies for minimization of measurement error • Found high accuracy through random sampling of audits MMAT: 100 %
Johnson (2010) [42] To determine whether self-reported receipt of lifestyle advice from a health care provider is lower among outpatient cardiac rehabilitation (OCR) non-attendees and non-referred patients compared to OCR attendees	In 2002–2007, % of patients receiving lifestyle advice from GPs: Advised to increase physical activity 71 % Advised to follow a modified fat diet 55 % Advised to quit smoking out of patients who smoked in last 6 months 88 %	Recommended that referred patients who do not attend CR be identified by their GP and encouraged to participate in home-based CR	• 65 % consented to inclusion • Consenters more likely to be male and undergo CABG • Analysis based on self-report: patient recall 5 months post-discharge • Large sample size: used Hunter New England Heart and Stroke Registry • Potential response bias • May underestimate appropriate advice • Considerable missing data MMAT: 100 %
Looi (2011) [28] To measure adherence to evidence-based ACS medications post-CABG	In 2006–2007, at 3 years post-CABG, % of medication usage by patients: Aspirin 83 % βB 62 % ACEi/ARB 43 % Statins 72 % Major adverse cardiological events (6.2 %/year): 3 UA, 4 NSTEMI, 6 HF, 5 deaths	Secondary prevention medication usage in ACS patients undergoing CABG was disappointingly low at discharge and worse at follow-up	• 86 % response rate • Association between cardiac events and low adherence to cardiac medication was not statistically assessed MMAT: 75 %
Mudge (2001) [33] To measure prescription of lipid-lowering drugs on discharge, and patient adherence at follow up	In 1998–1999, at 6–18 months post-ACS, patient status in lipid management: Did not have lipid measurements 10 % Of patients not prescribed LLD at discharge, patients who did not receive LLD prescription from GP 70 % Of those prescribed LLD on discharge, patients who remained on the treatment at GP follow up 88 %	Identified suboptimal lipid documentation with poor communication across hospital-community interface, poor ongoing monitoring and dosage adjustment	• No inferential statistics reported • Follow-up information incomplete • Self-report likely to overestimate compliance MMAT: 100 %
Reddy (2008) [39] To assess the extent to which evidence-based guidelines have influenced medical practice with respect to their experiences in depression assessment and management	Insights from surveys and interviews with GPs: • Little consistency among health professionals on how best to identify and manage depression • Few GPs asked patients about depression, regardless of patients’ depression score	• Wide distribution of guideline-related information was not effective in improving depression management • No agreement on appropriate time and provider for depression screening	• Published short report provides little detail regarding study design and quality appraisal • Study time frame not reported MMAT: n/a
Rushford (2007) [36] To assess patient recall of risk factor behaviour modifying intervention at discharge, 2, 4 and 12 months	Insights from study at 12 month follow-up: • CR referral is correlated with attendance • 8 % of women reported wanting more lifestyle advice	Limited advice provided on lifestyle (especially on diet & physical activity) to women who were obese or inactive. Older women less likely to recall receiving information Health staff need training in information delivery and communication skills	• Response rate 79 % • Good reasons for exclusion • Detailed assessment of recall on many areas of lifestyle • No details on how the initial patient education was conducted or its content MMAT: 100 %
Schrader (2005) [41] To evaluate the effect on depressive symptoms in cardiac patients of patient-specific advice to general practitioners regarding management of comorbid depression	In 2000–2001, in a randomized controlled trial: • The intervention had little effect on moderate to severe depression at 12 months • Telephone call to GP from psychiatrist led to a significant decrease in proportion of patients with moderate to severe depression • Multidisciplinary enhanced Primary Care case conference not effective (and difficult to implement)	Recommended screening of hospitalised cardiac patients for depression ansd providing targeted advice to their GPs	• No information on what management plans were actually delivered by GPs and no information on antidepressant prescription and service utilisation • Follow-up below 80 % (78.5 %) with differential non-reponse in younger separated/divorced patients and smokers • Allocation concealment unclear MMAT: 50 %
Schulz (2000) [37] To identify factors associated with and predicting attendance of post-MI patients at CR program	In 1993–1996, ~3.5 years post-MI: • 73.4 % referred to CR • Majority (72 %) of non-attenders were not referred to CR • Non-referral was significantly associated with non-attendance • Attendance significantly associated with referral	Being older, living farther away, living alone and not having private transport wre associated with CR non-attendance Referral to CR also predicted attendance	• 69 % response rate • Strengths and limitations of study well identified • Have not defined completion other than to offer second dropout rate of 36 % if attended 6 or fewer sessions MMAT: 75 %
Scott (2004) [31] To optimise care of patients with ACS and CHF through a QI intervention across two sectors (hospital and GP) of healthcare	In 2000–2002, at 3 months post-ACS, % of medications prescribed to patients: Aspirin: (baseline) 82 % (intervention) 89 % Aspirin continuation in those prescribed at discharge: (baseline) 84 % (intervention) 92 % βB continuation among those prescribed at discharge: (baseline) 76 % (intervention) 85 %	Implementing systems of decision support, targeted provider education & performance feedback, patient self-management and hospital-community integration improved patient care, particularly when directly controlled by individual clinicians (e.g., prescribing)	• Not possible to attribute specific process-of-care changes to specific QI initiatives within a multifaceted program • Only significant results reported MMAT: 50 %
Toms (2003) [34] To determine whether Phase II outpatient CR participants are more successful at achieving cardiac risk factor targets than non-participants at follow-up post-MI	In 2003, at 18–36 months post-MI: • Of 36 included CR non-participants (NP), 53 % not referred by doctor • CR participants less likely to have total cholesterol > 6.5 mmol/L • Fewer non-participants were receiving cholesterol lowering medication • In both groups, approximately 50 % did not achieve target total cholesterol (≤4.5 mmol/L) • CR participants more likely to be on lipid modifying treatment • More CR participants exercised regularly • Failure to achieve blood pressure and weight control similar in both groups • Small numbers continued smoking in both groups but insufficient sample size to assess statistical significance • More CR participants had returned to work (92 % vs. 78 %) but not statistically significant even after adjusting for age	Those attending CR had better long term outcomes, exercising more and more achieving the goal of a TC ≤6.5 mmol/l	• Participants resided within 40 km of Canberra therefore geography less of an issue • Used TC to assess lipids, not LDL • Highlight need for data collection • Low response rate (51 %) • Limitations of study recognised • Selection bias likely in those attending CR MMAT: 75 %
a. Wachtel (2008) [29] b. Wachtel (2008) [40] To determine assessment of lifestyle and behavioural risk factors in post-MI patients in hospital and at GP follow-up in a rural region of South Australia	In 2004–2005: • Population was 78 % male • One Aboriginal/Torres Strait Islander patient (2 %) • Majority of patients did not receive an intervention for risk factors • 5/11 (45 %) patient smokers received quit advice, one prescribed NRT • Higher proportion of patients received lifestyle interventions in GP practice than hospital setting, however, with the exception of smoking this accounted for 7 % of patients • 16/34 (47 %) patients had BMI assessed • 11 were overweight/obese of whom 2 (18 %) received weight loss advice	GPs generally increased prescribing of evidence based medications from time of discharge Major gap in CR and secondary prevention management of ACS patients in rural South Australia	• No documentation of special/additional services for ATSI population • Lifestyle and behavioural risk poorly documented except smoking status (76 %) and hypertension and diabetes (82 % and 78 %) • Low response rate MMAT: 50 %
a. Wai (2012) [20] b. Peterson (2012) [32] To improve the management of ACS at the point of hospital discharge, across the continuum of care	In 2009, at a median of 96-day- follow-up (range 49–204): • 48 % reported using 4 evidence-based medications (EBMs), with a significant decrease in anti-platelet agents, statins, β blockers and all 4 EBMs • 67 % recalled referral to CR of whom 33 % completed CR and 21 % were still attending CR • 731 GPs (47 % of patient-nominated GPs) participated in survey • 77 % received a discharge summary for patients with ACS at a median time of 3 days (0–41 days) after discharge • Of these 88 % contained a list of prescribed medications; 81 % included dose titration and duration of therapy and 55 % contained details of ongoing risk management • 65 % of GPs rated the quality of information as ‘very good’ to ‘excellent’ • 6 % increase in communication of ACS management plan to GP • 18 % increase in patients with documentated chest pain action plan	Targeted educational intervention can improve management of patients post-ACS Improvements evident in: • Evidence based prescribing • Communication between patient/carer 7 GP • Referrals to CR	• Accuracy of sample representation not documented • Based on medical record documentation and GP survey • Potential for Hawthorne effect • Low response rate of eligible GPs MMAT: 75 %

*ACEi/ARB* angiotensin-converting enzyme inhibitor/angiotensin-II receptor blocker, *ACS* acute coronary syndrome, β*B* beta-blockers, *BMI* body mass index, *CABG* coronary artery bypass grafting, *CR* cardiac rehabilitation, *DAPT* dual antiplatelet therapy, *EBM* evidence-based medication, *GP* general practitioner, *GTN* glycerol trinitrate, *HDL* high-density lipoprotein, *LDL* low-density lipoprotein, *LLD* lipid-lowering drugs, *MMAT* Mixed Methods Appraisal Tool, *MI* myocardial infarction, *NSTEMI* Non-ST elevation myocardial infarction, *NZ* New Zealand, *PCI* percutaneous coronary intervention, *QI* quality improvement, TC total cholesterol, *UA* unstable angina

### Pharmacotherapy

Six studies explored various aspects of pharmacological management of ACS in primary care. One recent Australian observational study reported outcomes of patients (*n* = 12813) following a percutaneous coronary intervention (PCI), the majority in the context of ACS [26]. In 2010, 75 % of patients were taking ≥ 4 classes of drugs at 12 month follow-up. The study also analysed trend data from 2005 to 2010 and found an increase over time in receipt of all drug classes investigated (*p* < 0.01). Finally, the same study found that females and patients aged > 75 years had significantly lower rates of medication usage than males and younger patients. Medication use was determined either by patient report or record review. An observational study conducted in New Zealand [27] reported similar rates of medication use at 3-year follow-up (*n* = 112). However, another New Zealand study reported lower rates of medication use in a cohort of coronary artery bypass grafting (CABG) patients (*n* = 109) at three-year follow-up [28]. Wachtel et al. [29] conducted a retrospective analysis of hospital and GP medical records (*n* = 34 patients), and found that GPs generally increased the prescription of evidence-based medications compared to prescription rates at hospital discharge.

Two interventional studies used medication prescription rates at GP follow-up as a clinical indicator to evaluate programs designed to improve post-ACS care [30, 31]. Hickey et al. [30] examined a quality improvement initiative known as the Brisbane Cardiac Consortium (BCC). The intervention included recurrent GP performance feedback from researchers in addition to GP liaison with hospitals regarding patient management. Though the study showed significant improvements in some clinical indicators, it did not report a significant change in prescription rates of medications in primary care follow-up post-intervention (*n* = 89 and *n* = 104 for 3- and 6- month follow-up, respectively). Scott et al. [31] examined the efficacy of a multi-faceted intervention that included clinical decision support, educational interventions, regular performance feedback, patient self-management strategies, and hospital-community integration. The study found greater prescription rates of aspirin at 3 months post-discharge in intervention patients compared to patients that received usual care (*p* = 0.05), and high rates of aspirin (*p* = 0.03) and β-blocker (*p* = 0.05) continuation among those prescribed these medications at discharge (*n* = 344). The intervention described in Scott et al. [31] also received generally positive feedback from patients.

In addition, Wai et al. [20] reported baseline results of medication use across Australia before a quality improvement initiative. This study reported that only 48 % of patients used four or more evidence-based drugs at a median of 96-day follow-up (*n* = 1319). When medication was stopped post-discharge, “the GP stopped it” was a major reason cited for discontinuation. The subsequent study to these baseline findings [32] did not report any medication usage rates in follow-up.

Two studies examined rates of lipid-lowering therapy (LLT) use in primary care follow-up. Mudge et al. [33] found that 66 % of post-ACS patients were on LLT 6–18 months post-discharge, and that 18 % of post-ACS patients with cholesterol levels over target did not receive LLT (*n* = 282). Toms et al. [34] measured a 50 % LLT prescription rate 18–36 months post-discharge, and that more than 50 % of study participants had total serum cholesterol levels above target (*n* = 93).

### Behaviour change

Five studies described behaviour change related to ACS in primary care. Gallagher et al. [35] studied an all-female population to determine predictors of completion of CR (*n* = 196). This descriptive study surveyed patients 12 weeks post-discharge, and found that two-thirds of women were referred to CR, and that CABG patients were more likely to be referred to CR than were myocardial infarction (MI) patients (though statistical analysis was not provided).

Rushford et al. [36] examined Australian female ACS patients and reported that recall by patients of CR referral by physicians, physiotherapists, nurse practitioners, or dietitians was correlated with attendance (*p* = 0.001) in this cohort (*n* = 212). Schulz et al. [37] found CR referral to be the single biggest influencer of attendance, and also found that being younger (*p* = 0.032) or married (*p* = 0.03) or living with a partner (*p* = 0.05) made patients more likely to be referred to CR (*n* = 79). Gender was not determined by this study to be a factor influencing CR attendance. Toms et al. [34] compared CR participants with non-participants in a observational study, and found that non-participants cited non-referral most commonly as the reason for their non-attendance, and that younger patients were more likely to be participate in CR. In results from a 2012 Australia-wide quality improvement study [32], both GPs and patients reported a 6 % increase in CR referral by GPs post-educational intervention (*n* = 636, p-values = 0.05 and 0.001 for GPs and patients, respectively).

The same interventional study examined the effect of an education intervention across the continuum of care on patients’ possession of an ACS management plan [32], which is an evidence-based guideline recommended for all post-ACS patients [5]. Compared to baseline, Peterson et al. [32] found that more patients had documented ACS management plans (*n* = 1589, *p* = 0.01), and of these, more plans contained a chest pain action plan (*n* = 1383, *p* < 0.0001). This intervention also increased communication of this management plan to the GP (*n* = 1589, *p* = 0.0001).

### Psychological assessment

Psychological management in primary care was described in four studies. Fernandez et al. [38] described significant levels of depression, anxiety and documented stress in an observational study of patients 12–18 months after a percutaneous coronary intervention (PCI) (*n* = 202). Reddy et al. [39] described a lack of consistency in GP adherence to a protocol regarding depression screening, and that in a study of GPs, “about half” of the GPs were prepared to prescribe antidepressants (*n* = 18). In this same study, few GPs asked patients about depression, despite having received information about their patient’s depression score. Wachtel et al. [40] found that no ACS patients received any relevant behavioural interventions by a GP (*n* = 55), and no ACS patients received a social support or living condition intervention by a GP (*n* = 45). A randomised controlled trial conducted by Schrader et al. [41] documented that a GP intervention involving a telephone consult with a psychiatrist was the most effective psychological intervention and resulted in a reduced relative risk of having moderate to severe depression (n-237, 95 % CI). The same study found that non-consent to study procedures was associated with being older (*p* < 0.001) and female (*p* < 0.001).

### Lifestyle management

Seven studies discussed management of lifestyle and behavioural risk factors. Fernandez et al. [38] documented that 46 % of female and 25 % of male patients had two or more modifiable risk factors one year after a PCI (*n* = 202). The same study found that patients underestimated their possession of risk factors: for both hypertension and hypercholesterolaemia, the portion of patients that reported these conditions were 9 % and 40 % lower than the portion of patients who had blood pressure and total cholesterol levels above target, respectively [38].

One study in Canberra reported that 12 % of CR participants, 53 % of CR non-participants, and 28 % of all studied patients still smoked 18–36 months after an MI (*n* = 93) [34]. Another observational study in Sydney [38] reported that 15 % of PCI patients were active smokers at 12- to 18- month follow-up post-PCI. A cross-sectional study in Auckland (*n* = 202) [27], found that 52 % of smokers had quit 3 years post-discharge from an ACS after an implicit intervention by the GP, resulting in an 11 % decrease in smoking in the study population.

A cohort study conducted by Wachtel et al. [29] reported that 45 % of patients received a smoking intervention in their GP clinics, while none received this intervention in the hospital setting (*n* = 34). A larger cross-sectional study [42] found that 88 % of post-ACS smokers received advice to quit (*n* = 674). One cross-sectional study [30] reported a significant improvement of smoking cessation at 3- and 6- month post- ACS event (*n* = 89 and *n* = 104, respectively) after a quality improvement program which included an education intervention targeting hospitals and GPs (*p* ≤ 0.05 for both).

A qualitative study [43] which described how Australian smokers and ex-smokers viewed the role of their GPs post-ACS found that many of the participants expressed a negative reaction to GP advice about smoking cessation, especially when it was unsolicited (*n* = 41). Some participants expressed that smoking advice was hard to receive when they were unwell or frightened. Patients in the study expressed feeling distressed when GPs attributed all their health problems to smoking, and some admitted to lying to their GP about quitting. Participants often described the manner in which GPs spoke to them about smoking cessation as significant: doctors who had quit smoking or had personal experiences with smoking were more likely to be persuasive.

Other studies described management of physical activity in primary care. The observational study conducted by Fernandez et al. [38] documented that 12 % of patients performed no physical activity 12–18 months after a PCI (*n* = 202), while the Toms et al. study [34] found that 65 % of MI patients exercised less than 3 times a week 18–36 months post-MI (*n* = 93). Ford et al. [27] found that in New Zealand, 47 % patients exercised 4 or more times a week 3 years after their ACS event (*n* = 112). Johnson et al. [42] reported that 76 % of patients were told to increase physical activity by a primary care professional (*n* = 4330), while another study [40] found that only 3 % of patients had an intervention in their GP clinic regarding physical activity (*n* = 34).

Fernandez et al. [38] reported 18 % of patients had hypercholesterolaemia 12–18 months after a PCI (*n* = 200). A cross-sectional study [27] showed a 0.8 mmol/L decrease in total cholesterol levels 3 years after ACS discharge (*P* < 0.001) compared to pre-PCI values (*n* = 112). One study found that 60 % of patients received advice in a primary care setting to follow a modified fat diet (*n* = 4347) [42] while another found that only 6 % of patients (*n* = 34) reported receiving advice regarding their dietary habits in a GP clinic [40]. The same study also describes that 7 % (*n* = 29) of patients reported receiving a GP intervention about being obese or overweight, while 3 % (*n* = 33) reported receiving an intervention for alcohol intake.

Besides the four categories outlined by the National Heart Foundation of Australia (NHFA), one study also examined hospital communication as a barrier to primary care management of ACS. As reported by Wai et al. [20], only 77 % of GPs (*n* = 731) reported receiving discharge summaries of ACS patients from hospitals at baseline. In addition, not all discharge summaries included prescribed medications (88 %) and risk factor management details (55 %). Only 65 % participating in the GP survey considered the quality of hospital information provided as “very good” or “excellent”. A subsequent educational intervention did not significantly improve the quality of these discharge summaries [32].

## Discussion

Though adherence to post-ACS management guidelines varied across studies, it is clear that there is much room for improvement in optimising follow-up care following discharge after an acute cardiac event. Reducing morbidity and mortality through adequate secondary prevention would also be financially prudent as it would reduce costs to the health care system. De Guyter and colleagues undertook a cost benefit analysis over a 10 year period which estimated substantial economic and social impacts of increasing the uptake of cardiac rehabilitation and secondary prevention [44]. Compared with a base case of 30 % uptake, increasing uptake of CR to 50 % (scenario 1) or 65 % (scenario 2) gave a benefit cost ratio of 5.6 and 6.8 which translated to net financial savings of $46.7 million (scenario 1) and $86.7 million (scenario 2) and a reduction in Disability Adjusted Life Years of 21,117 to 37,565 compared with the base. Given the pre-eminent role of primary health care in supporting patient care outside of the hospital, this component of the health care system has a role in secondary prevention that is essential to improving outcomes following coronary events and in minimising unnecessary health care costs, and hence the need to examine how well this is being undertaken.

### Knowledge of post-ACS GP management

This review is unique in its summary of the knowledge base of primary care of coronary artery disease post-ACS in Australia and New Zealand. The 19 peer-reviewed publications show there is fragmented understanding of post-ACS care by GPs in Australia and New Zealand. Since the studies are diverse in study design, quality, analytical methods, setting, and time frame, it is difficult to discern any emerging patterns. However, some strengths and weaknesses do arise.

Studies of medication prescription rates post-ACS report mostly high rates of antiplatelet therapy prescription [20, 26, 27, 31], with one study reporting an encouraging positive trend in medication prescription over time [26]. However, the prescription rates of other evidence-based medications demonstrate room for improvement, especially angiotensin-converting enzyme inhibitor/angiotensin-II receptor blockers (ACEi/ARBs) and glycerol trinitrate (GTN) spray [27, 28]. Out of two interventional studies aiming to improve medication prescription rates, only one reported a significant positive impact [31]. The limited number and success of interventional studies and the diverse range of medication prescription rates (as described in Table 3) highlight the need for further research in ways to improve medication prescription by GPs in primary care.

Studies examining CR paid little attention to the role of GPs in influencing CR attendance: only one intervention sought to improve CR referral by GPs [32]. This is a matter of concern, especially since studies reported low referral rates [35, 37] and a strong relationship between referral and attendance [36]. Since studies highlighted that female [35], elderly, and single [37], patients are less likely to be referred to CR, interventions that focus on the needs of these special populations are indicated. In addition, since an educational intervention was found to have a significant positive impact on GP referral to CR and to creation of ACS management plans [32], the wider implementation of this quality improvement initiative has the potential to improve outcomes. Johnson and colleagues strongly recommended that non-referred patients be identified by their GP and be referred to CR and those who were referred but did not attend be identified and encouraged to participate in an alternative home-based CR program [42].

Although Fernandez et al. [38] clearly highlighted the need for psychological management of patients with ACS, there is little consensus or study of psychological assessment in primary care. Two descriptive studies found a complete lack of intervention [29] and inconsistent beliefs regarding depression screening in the primary care setting [39], highlighting an urgent need for more research to enhance primary care psychological management of coronary disease. Both studies also used small sample sizes, so research studying psychological assessment on a larger scale would be advantageous. The single randomised controlled trial that examined depression management found that a telephone consultation with a psychiatrist was effective [41], suggesting that larger scale implementation of this practice in primary care could be effective.

In studies where lifestyle management was studied, the possession of multiple modifiable risk factors post-ACS patients was common [33, 34, 38], while GP advice or intervention regarding these risk factors was inconsistent [27, 42] and sometimes severely lacking [40]. One promising interventional study found success in increasing GP interventions regarding smoking cessation post-ACS [30], but the success of these interventions in actually causing patients to quit smoking is still unclear. A qualitative study highlighted the complexity of GP involvement in smoking cessation [43], as advice regarding smoking cessation was not always regarded positively, and opinions were inconsistent regarding the productivity of such advice.

Hypercholesterolaemia management through modified diet advice and LLT was generally lacking [38, 42]. In addition, though body mass index (BMI) was not widely addressed, two studies reported alarming rates of obese and overweight post-ACS patients [29, 38], while advice from GPs regarding physical activity was imperfect [42]. Little is known about GP management and advice regarding alcohol intake in the context of post-ACS care. Besides depression management and a single study of a diabetic cohort, included publications failed to address in detail the complexities of handling ACS patients with other relevant comorbidities. Research in this area would assist a GP in adequately managing such complex cases. Surprisingly, there were few studies for whom the provision and effectiveness of lifestyle management advice was assessed, and these had small sample sizes. Concerns regarding the efficacy of GP advice in risk factor modification have the potential to influence the rate at which GPs deliver such advice, and so the development of robust strategy regarding GP advice to reduce patient possession of modifiable risk factors would be beneficial. This is an area where practice nurses with chronic disease expertise and ancillary allied health practitioners working within a general practice setting could offer opportunities for improved patient advice and outcomes.

Wai et al. [20] highlighted a need for increased communication between hospitals and GPs post-ACS. A subsequent quality intervention across the continuum of care was promising [32], and has the potential to improve multiple areas of evidence-based management of post-ACS care.

### Special populations

While Indigenous populations are identifiable as collectively having poorer health status in Australia and New Zealand [45], no published studies focused exclusively on the primary care management of Indigenous populations that have suffered an ACS. This is despite documentation that Indigenous populations of both nations face a disproportionate cardiovascular disease burden and experience culture-specific barriers to care [46]. Besides Indigenous populations, no Australian studies examined post-discharge ACS management in any non-Indigenous minority populations, although these groups were better addressed in publications from New Zealand [20, 28, 32]. Rural populations face unique barriers to care, and yet only five studies examined different aspects of patient care and follow-up in this setting. This is noteworthy because a significant portion of both Australia and New Zealand’s populations reside in rural areas. In addition, while many included studies reported patient populations that were overwhelmingly male, there was limited analysis of gender-based differences in primary care management of ACS. This is significant because post-ACS care decisions have historically been gendered. Overall, though age- and gender-related data was consistently collected, limited analysis of this data was reported. Overwhelmingly, studies followed patients from hospital rather than focusing on GP care. Published literature was also lacking in qualitative data, which could expose underlying attitudes and beliefs that affect patient care.

### Study quality

Out of 17 unique studies, 13 received MMAT scores ≥ 75 %, indicating that studies were of generally good quality. No study received a score below 50 %, though the MMAT score of one study [39] could not be assessed, since its study protocol was not fully reported.

### Strengths and limitations

This review highlights the challenges of and potential opportunities for improvement in post-ACS management in primary care in Australia and New Zealand. As ACS management, as specified by the NHFA and the CSANZ [5] and supplemented by the Cardiovascular Therapeutic Guidelines [6], covers such a broad range of treatment and care, this study is unique reporting on the status of primary care management and research in this area. This review is strengthened by its thorough literature search of multiple databases, and by the quality appraisal of publications in the MMAT format.

Several factors limit the definitive conclusions that can be drawn from the review. In the literature selection process, the possibility exists that relevant article(s) were not identified by the literature search because of gaps in implemented database search strings, although search strings for each database were developed with careful consideration and input from multiple authors, they were not proofread by any third-party assistant or librarian. The decision to limit this review to peer-reviewed publications additionally creates the potential of publication bias [47], especially since studies of populations often neglected by peer-reviewed publications, like Indigenous or rural populations, may be conducted by organisations that lack the financial means to publish academically.

The mixed-methods nature of the publications obtained from the literature search limited the nature of analysis. A meta-analysis was impossible, because of the inclusion of studies that were qualitative, descriptive, and non-interventional. In addition, studies were often limited in the amount of data that was publicly available: studies sometimes only published significant findings or short reports rather than exhaustive research papers. This data limitation was further complicated by the fact that the aim or research question of included studies often differed drastically from the aim of this review (Table 4). The number of publications also restricted the ability to draw conclusions about areas for improvements across the continuum of care. Studies that describe successful interventions may not apply in special population groups, but since special populations were largely not addressed, it is difficult to determine the success of interventions for them. The limited number of publications suggests that more follow-up data or research in this area is required.

The coverage of the subject matter of these publications was also restricted in regard to barriers and facilitators to primary care management of ACS, because the studies do not include patients that were prevented by some means from accessing a GP in the first place.

## Conclusions

Primary care management of post-ACS patients is proven to increase the quality of patients’ lives, and reduce their risk of a secondary cardiac event and healthcare system costs of rehospitalisation. Understanding management in primary care and identifying gaps is essential to improving the quality of care for a common, serious, cardiovascular condition where unnecessary readmissions can be avoided. Given high rates of CVD, relatively few papers were identified regarding management in primary health care settings after an acute coronary syndrome event. This is surprising given the importance of support for patients at this time, specifically around adherence to evidence based medications and adopting a healthy lifestyle in order to reduce the chances of a recurrence. There were few interventional studies, so further research of ways to improve quality of care is clearly indicated. Future study should include efforts to improve the quality of care of special population groups must be customised to their particular needs [46]. It is clear that greater integration of hospital and GP management in the form of detailed discharge summaries and communication of management plans would allow for more effective patient care.

## Additional file

Additional file 1: Search strategy and keywords. Terms used in the literature search. (DOCX 27 kb)

## Abbreviations

ACEi/ARB: Angiotensin-converting enzyme inhibitor/angiotensin-II receptor blocker
ACS: Acute coronary syndrome
BMI: Body mass index
CABG: Coronary artery bypass grafting
CR: Cardiac rehabilitation
CSANZ: Cardiac Society of Australia and New Zealand
CVD: Cardiovascular disease
DAPT: Dual antiplatelet therapy
EBM: Evidence-based medication
HDL: High-density lipoprotein
LDL: Low-density lipoprotein
LLT: Lipid-lowering therapy
MeSH: Medical subject headings
MI: Myocardial infarction
MMAT: Mixed methods appraisal tool
NHFA: National Heart Foundation of Australia
NSTEMI: Non-ST-segment elevation myocardial infarction
NZ: New Zealand
PCI: Percutaneous coronary intervention
QI: Quality improvement
RCT: Randomised controlled trial
STEMI: ST-segment elevation myocardial infarction
βB: Beta-blocker
