# Supplementary material for: Evidence-based interventions in primary care following acute coronary syndrome in Australia and New Zealand: a systematic scoping review
Source: BMC Cardiovasc Disord. 2016 Nov 9;16:214. doi: 10.1186/s12872-016-0388-y (PMC5103388; doi:10.1186/s12872-016-0388-y)
Supplement: Additional file 1: — Search strategy and keywords. Terms used in the literature search. (DOCX 27 kb) [file 12872_2016_388_MOESM1_ESM.docx]

Additional file 1 Search Strategy and Keywords

| Search | |  |  | Search String |  | Citations |
| --- | --- | --- | --- | --- | --- | --- |
| 1 | Targets terms related to Acute Coronary Syndrome | |  | Acute Coronary Syndrome[mh] OR Unstable Angina[mh] OR Myocardial Infarction[mh] OR acute coronary[tw] OR myocardial infarction*[tw] OR STEMI*[tw] OR NSTEMI*[tw] OR unstable angina*[tw] OR coronary thrombosis[tw] OR coronary thromboses[tw] OR cardiac arrest*[tw] OR heart arrest*[tw] OR cardiac incident*[tw] OR acute heart disease*[tw] OR ((Hospitalization[mh] OR hospitali*[tw] OR admit*[tw] OR admission*[tw] OR acute[tw] OR unstable[tw]) AND (coronary[tw] OR ischemic heart[tw] OR ischaemic heart[tw] OR myocardial isch*[tw])) |  | 295436 |
| 2 | Targets terms related to Patient Discharge | |  | Patient Discharge[mh] OR Patient Discharge Summaries[mh] OR Continuity of Patient Care[mh] OR discharge*[tw] OR post-discharge*[tw] OR postdischarge*[tw] OR care continuity[tw] OR continuity of care[tw] OR care continuum [tw] OR continuum of care[tw] OR continuation*[tw] OR care transition*[tw] OR transition in care[tw] OR transitions in care[tw] OR transition of care[tw] OR transitions of care[tw] OR transitional care[tw] OR follow up*[tw] OR postacute[tw] OR post-acute[tw] OR post acute[tw] OR posthospital*[tw] OR post-hospital*[tw] OR post hospital*[tw] OR postmyocard*[tw] OR post-myocard*[tw] OR post myocard*[tw] OR after hospitali*[tw] OR following hospitali*[tw] OR care pathway*[tw] OR pathway of care[tw] OR pathways of care[tw] |  | 1211783 |
| 3 | Targets terms related to Primary Care | |  | Physicians, Primary Care[mh] OR General Practice[mh] OR Physicians, Family[mh] OR Primary Health Care[mh] OR general practitioner*[tw] OR primary care physician*[tw] OR family practice*[tw] OR family practitioner*[tw] OR general practice*[tw] OR primary practice*[tw] OR primary care[tw] OR primary health care[tw] OR family physician*[tw] OR family doctor*[tw] |  | 241987 |
| 4 | Targets terms related to cardiac rehabilitation | |  | cardiac rehabilitation*[tw] OR cardiovascular rehabilitation*[tw] |  | 4331 |
| 5 | Targets terms related to secondary prevention | |  | Secondary Prevention[mh] OR secondary risk reduction*[tw] OR secondary prevention*[tw] |  | 27157 |
| 6 | Combines Acute Coronary Syndrome, Primary Care, and Patient Discharge terms | |  | #1 AND #2 AND #3 |  | 987 |
| 7 | Combines acute coronary syndrome with cardiac rehabilitation and Patient Discharge terms | |  | #1 AND #2 AND #4 |  | 737 |
| 8 | Combines acute coronary syndrome with secondary prevention and Patient Discharge terms | |  | #1 AND #2 AND #5 |  | 1609 |
| 9 | Combines previous to provide articles pertaining to primary care post-discharge management of ACS | |  | #6 OR #7 OR #8 |  | 3045 |
| 10 | Limit to English | |  | #9 AND eng[la] |  | 2672 |
| 11 | Limit to journal articles | |  | #10 AND journal article[pt] |  | 2640 |
| 12 | Limit to articles after 2000 | |  | #11 AND 2000:2015[dp] |  | 2127 |
| 13 |  | |  | **Australia*[tw] OR Australasia*[tw] OR New Zealand*[tw] OR ANZ[tw] OR Western Australia*[tw] OR Northern Territory*[tw] OR Queensland*[tw] OR South Australia*[tw] OR New South Wales*[tw] OR Victoria*[tw] OR Tasmania*[tw] OR Sydney*[tw] OR Melbourne*[tw] OR Brisbane*[tw] OR Perth*[tw] OR Adelaide*[tw] OR Auckland*[tw] OR Wellington*[tw] OR Christchurch*[tw] OR Dunedin*[tw]** | BH |  |
| 14 |  | |  | 0001027 [jid] OR 0001061 [jid] OR 0015376 [jid] OR 0021406 [jid] OR 0032450 [jid] OR 0047441 [jid] OR 0111052 [jid] OR 0112726 [jid] OR 0117710 [jid] OR 0135232 [jid] OR 0135240 [jid] OR 0137162 [jid] OR 0144065 [jid] OR 0175411 [jid] OR 0201723 [jid] OR 0222472 [jid] OR 0227625 [jid] OR 0235674 [jid] OR 0245020 [jid] OR 0245021 [jid] OR 0251041 [jid] OR 0254511 [jid] OR 0254561 [jid] OR 0263662 [jid] OR 0322433 [jid] OR 0326701 [jid] OR 0332637 [jid] OR 0342017 [jid] OR 0347221 [jid] OR 0365543 [jid] OR 0366537 [jid] OR 0367666 [jid] OR 0370074 [jid] OR 0370357 [jid] OR 0370612 [jid] OR 0370613 [jid] OR 0370614 [jid] OR 0370615 [jid] OR 0370616 [jid] OR 0372377 [jid] OR 0372637 [jid] OR 0373115 [jid] OR 0377430 [jid] OR 0400714 [jid] OR 0401065 [jid] OR 0401067 [jid] OR 0405374 [jid] OR 0414755 [jid] OR 0416662 [jid] OR 0425076 [jid] OR 0425725 [jid] OR 0427360 [jid] OR 100883647 [jid] OR 100883685 [jid] OR 100886002 [jid] OR 100886426 [jid] OR 100886721 [jid] OR 100888004 [jid] OR 100888170 [jid] OR 100888373 [jid] OR 100891857 [jid] OR 100895290 [jid] OR 100896531 [jid] OR 100910109 [jid] OR 100955225 [jid] OR 100955338 [jid] OR 100956805 [jid] OR 100958810 [jid] OR 100963739 [jid] OR 100966102 [jid] OR 100966143 [jid] OR 100966196 [jid] OR 100966232 [jid] OR 100966267 [jid] OR 100966383 [jid] OR 100966385 [jid] OR 100966387 [jid] OR 100966388 [jid] OR 100966389 [jid] OR 100966390 [jid] OR 100966394 [jid] OR 100967044 [jid] OR 100967186 [jid] OR 100967485 [jid] OR 100967660 [jid] OR 100967746 [jid] OR 100967755 [jid] OR 100967856 [jid] OR 100967888 [jid] OR 100967906 [jid] OR 100967934 [jid] OR 100967941 [jid] OR 100968725 [jid] OR 100968839 [jid] OR 100969013 [jid] OR 100969096 [jid] OR 100969251 [jid] OR 100969253 [jid] OR 100969255 [jid] OR 100969260 [jid] OR 100969465 [jid] OR 100969544 [jid] OR 100969794 [jid] OR 100969864 [jid] OR 100969889 [jid] OR 100969910 [jid] OR 100970115 [jid] OR 100970526 [jid] OR 100970528 [jid] OR 100970531 [jid] OR 100970533 [jid] OR 100970536 [jid] OR 100970539 [jid] OR 100970736 [jid] OR 100970807 [jid] OR 100971272 [jid] OR 100971365 [jid] OR 100971452 [jid] OR 100971533 [jid] OR 100971934 [jid] OR 100971981 [jid] OR 100972137 [jid] OR 100972164 [jid] OR 100972175 [jid] OR 100972189 [jid] OR 100972330 [jid] OR 100972491 [jid] OR 100972636 [jid] OR 100972640 [jid] OR 100972652 [jid] OR 100972723 [jid] OR 100972724 [jid] OR 100972763 [jid] OR 100972848 [jid] OR 100973029 [jid] OR 100973097 [jid] OR 100973175 [jid] OR 100973180 [jid] OR 100973220 [jid] OR 100973286 [jid] OR 100973293 [jid] OR 100973351 [jid] OR 100973353 [jid] OR 100973394 [jid] OR 101082644 [jid] OR 101082692 [jid] OR 101084284 [jid] OR 101084402 [jid] OR 101084426 [jid] OR 101084957 [jid] OR 101085100 [jid] OR 101085123 [jid] OR 101085144 [jid] OR 101085195 [jid] OR 101085272 [jid] OR 101085493 [jid] OR 101085823 [jid] OR 101085985 [jid] OR 101085999 [jid] OR 101086397 [jid] OR 101086634 [jid] OR 101086683 [jid] OR 101087210 [jid] OR 101087290 [jid] OR 101087416 [jid] OR 101088140 [jid] OR 101088612 [jid] OR 101090771 [jid] OR 101091136 [jid] OR 101091397 [jid] OR 101091407 [jid] OR 101091408 [jid] OR 101091415 [jid] OR 101091427 [jid] OR 101092430 [jid] OR 101092457 [jid] OR 101092952 [jid] OR 101096027 [jid] OR 101121465 [jid] OR 101121542 [jid] OR 101121619 [jid] OR 101122643 [jid] OR 101123037 [jid] OR 101130873 [jid] OR 101132262 [jid] OR 101132974 [jid] OR 101132977 [jid] OR 101133131 [jid] OR 101135738 [jid] OR 101140527 [jid] OR 101142614 [jid] OR 101142623 [jid] OR 101143078 [jid] OR 101150311 [jid] OR 101150314 [jid] OR 101154140 [jid] OR 101154361 [jid] OR 101156268 [jid] OR 101162874 [jid] OR 101171505 [jid] OR 101171544 [jid] OR 101174626 [jid] OR 101174860 [jid] OR 101179386 [jid] OR 101181252 [jid] OR 101196148 [jid] OR 101199488 [jid] OR 101199824 [jid] OR 101213954 [jid] OR 101234237 [jid] OR 101234238 [jid] OR 101235428 [jid] OR 101235528 [jid] OR 101235568 [jid] OR 101240304 [jid] OR 101241430 [jid] OR 101242667 [jid] OR 101244624 [jid] OR 101245791 [jid] OR 101245797 [jid] OR 101247063 [jid] OR 101248265 [jid] OR 101248313 [jid] OR 101250502 [jid] OR 101250741 [jid] OR 101253281 [jid] OR 101255828 [jid] OR 101256319 [jid] OR 101256593 [jid] OR 101258149 [jid] OR 101261851 [jid] OR 101263847 [jid] OR 101264260 [jid] OR 101266131 [jid] OR 101266965 [jid] OR 101269487 [jid] OR 101270281 [jid] OR 101273115 [jid] OR 101273479 [jid] OR 101273480 [jid] OR 101273481 [jid] OR 101276050 [jid] OR 101278333 [jid] OR 101282742 [jid] OR 101288638 [jid] OR 101293234 [jid] OR 101300022 [jid] OR 101302699 [jid] OR 101302940 [jid] OR 101303127 [jid] OR 101309314 [jid] OR 101310097 [jid] OR 101312158 [jid] OR 101312647 [jid] OR 101312780 [jid] OR 101313377 [jid] OR 101320027 [jid] OR 101320232 [jid] OR 101321511 [jid] OR 101321512 [jid] OR 101322827 [jid] OR 101463741 [jid] OR 101466475 [jid] OR 101467187 [jid] OR 101467908 [jid] OR 101467911 [jid] OR 101469340 [jid] OR 101471195 [jid] OR 101471610 [jid] OR 101473729 [jid] OR 101474356 [jid] OR 101475745 [jid] OR 101475748 [jid] OR 101477548 [jid] OR 101477550 [jid] OR 101478447 [jid] OR 101479763 [jid] OR 101488505 [jid] OR 101489913 [jid] OR 101492420 [jid] OR 101501666 [jid] OR 101504280 [jid] OR 101504326 [jid] OR 101505540 [jid] OR 101506757 [jid] OR 101506777 [jid] OR 101509728 [jid] OR 101511022 [jid] OR 101512437 [jid] OR 101512633 [jid] OR 101512640 [jid] OR 101512684 [jid] OR 101512688 [jid] OR 101512691 [jid] OR 101512700 [jid] OR 101512706 [jid] OR 101512875 [jid] OR 101512929 [jid] OR 101513378 [jid] OR 101514106 [jid] OR 101514107 [jid] OR 101514108 [jid] OR 101514322 [jid] OR 101514560 [jid] OR 101514563 [jid] OR 101514834 [jid] OR 101515268 [jid] OR 101515487 [jid] OR 101515585 [jid] OR 101515668 [jid] OR 101515695 [jid] OR 101515943 [jid] OR 101516207 [jid] OR 101517658 [jid] OR 101518866 [jid] OR 101520219 [jid] OR 101521147 [jid] OR 101524060 [jid] OR 101524435 [jid] OR 101524471 [jid] OR 101525397 [jid] OR 101525768 [jid] OR 101525771 [jid] OR 101526749 [jid] OR 101528540 [jid] OR 101528691 [jid] OR 101529202 [jid] OR 101529205 [jid] OR 101530345 [jid] OR 101530425 [jid] OR 101530479 [jid] OR 101531415 [jid] OR 101531636 [jid] OR 101531698 [jid] OR 101531700 [jid] OR 101531842 [jid] OR 101531893 [jid] OR 101532800 [jid] OR 101533144 [jid] OR 101533685 [jid] OR 101533715 [jid] OR 101533734 [jid] OR 101533742 [jid] OR 101533820 [jid] OR 101534028 [jid] OR 101534797 [jid] OR 101535239 [jid] OR 101535328 [jid] OR 101535593 [jid] OR 101535812 [jid] OR 101535817 [jid] OR 101535920 [jid] OR 101535922 [jid] OR 101536107 [jid] OR 101537753 [jid] OR 101537767 [jid] OR 101538816 [jid] OR 101540514 [jid] OR 101540978 [jid] OR 101542736 [jid] OR 101542737 [jid] OR 101543449 [jid] OR 101543450 [jid] OR 101544775 [jid] OR 101544801 [jid] OR 101545287 [jid] OR 101549953 [jid] OR 101550186 [jid] OR 101550215 [jid] OR 101550216 [jid] OR 101550217 [jid] OR 101550884 [jid] OR 101551170 [jid] OR 101551288 [jid] OR 101552395 [jid] OR 101556207 [jid] OR 101558476 [jid] OR 101558564 [jid] OR 101559142 [jid] OR 101560564 [jid] OR 101561207 [jid] OR 101562656 [jid] OR 101562700 [jid] OR 101562709 [jid] OR 101564865 [jid] OR 101566036 [jid] OR 101566041 [jid] OR 101566043 [jid] OR 101566269 [jid] OR 101566276 [jid] OR 101570796 [jid] OR 101571840 [jid] OR 101576715 [jid] OR 101576821 [jid] OR 101578235 [jid] OR 101579785 [jid] OR 101579789 [jid] OR 101580722 [jid] OR 101584913 [jid] OR 101586945 [jid] OR 101587774 [jid] OR 101587980 [jid] OR 101592082 [jid] OR 101593783 [jid] OR 101595023 [jid] OR 101595026 [jid] OR 101595308 [jid] OR 101596730 [jid] OR 101598595 [jid] OR 101599229 [jid] OR 101600601 [jid] OR 101600744 [jid] OR 101601804 [jid] OR 101603822 [jid] OR 101603961 [jid] OR 101603964 [jid] OR 101605450 [jid] OR 101606818 [jid] OR 101607189 [jid] OR 101608351 [jid] OR 101608659 [jid] OR 101610218 [jid] OR 101613518 [jid] OR 101617983 [jid] OR 101619315 [jid] OR 1255204 [jid] OR 1260462 [jid] OR 1264321 [jid] OR 1264322 [jid] OR 15010200R [jid] OR 15120050R [jid] OR 15320210R [jid] OR 15420100R [jid] OR 15420110R [jid] OR 15420200R [jid] OR 15420250R [jid] OR 15420270R [jid] OR 15420280R [jid] OR 15420300R [jid] OR 15420320R [jid] OR 15420340R [jid] OR 16420690R [jid] OR 16520450R [jid] OR 17430040R [jid] OR 18030020R [jid] OR 18920750R [jid] OR 19110830R [jid] OR 19110850R [jid] OR 19940420R [jid] OR 20440350R [jid] OR 20440740R [jid] OR 21040400R [jid] OR 21410020R [jid] OR 23630080R [jid] OR 2984699R [jid] OR 2984823R [jid] OR 53010090R [jid] OR 7501266 [jid] OR 7501311 [jid] OR 7503099 [jid] OR 7505323 [jid] OR 7505560 [jid] OR 7505855 [jid] OR 7506112 [jid] OR 7506123 [jid] OR 7506124 [jid] OR 7507039 [jid] OR 7507190 [jid] OR 7507812 [jid] OR 7508031 [jid] OR 7513553 [jid] OR 7513650 [jid] OR 7600076 [jid] OR 7606849 [jid] OR 7611484 [jid] OR 7613464 [jid] OR 7613841 [jid] OR 7703966 [jid] OR 7703975 [jid] OR 7704025 [jid] OR 7704769 [jid] OR 7802207 [jid] OR 7805150 [jid] OR 7806536 [jid] OR 7808693 [jid] OR 7809869 [jid] OR 7908180 [jid] OR 7909724 [jid] OR 8006208 [jid] OR 8007200 [jid] OR 8100311 [jid] OR 8203690 [jid] OR 8204496 [jid] OR 8208130 [jid] OR 8214381 [jid] OR 8215797 [jid] OR 8218028 [jid] OR 8300517 [jid] OR 8406538 [jid] OR 8408620 [jid] OR 8409358 [jid] OR 8412297 [jid] OR 8501421 [jid] OR 8505423 [jid] OR 8508146 [jid] OR 8600620 [jid] OR 8606184 [jid] OR 8607909 [jid] OR 8609734 [jid] OR 8703442 [jid] OR 8706300 [jid] OR 8709214 [jid] OR 8709379 [jid] OR 8711141 [jid] OR 8802146 [jid] OR 8802712 [jid] OR 8803700 [jid] OR 8807312 [jid] OR 8807862 [jid] OR 8811037 [jid] OR 8811765 [jid] OR 8903009 [jid] OR 8907465 [jid] OR 8910066 [jid] OR 8911454 [jid] OR 8915229 [jid] OR 9002928 [jid] OR 9005421 [jid] OR 9011101 [jid] OR 9012781 [jid] OR 9012921 [jid] OR 9012965 [jid] OR 9015440 [jid] OR 9101419 [jid] OR 9102074 [jid] OR 9105166 [jid] OR 9106714 [jid] OR 9108419 [jid] OR 9113681 [jid] OR 9114114 [jid] OR 9204105 [jid] OR 9207852 [jid] OR 9212162 [jid] OR 9212404 [jid] OR 9213011 [jid] OR 9305903 [jid] OR 9306292 [jid] OR 9306673 [jid] OR 9310939 [jid] OR 9313253 [jid] OR 9313485 [jid] OR 9314927 [jid] OR 9317904 [jid] OR 9421087 [jid] OR 9421464 [jid] OR 9421515 [jid] OR 9425670 [jid] OR 9426100 [jid] OR 9426344 [jid] OR 9430670 [jid] OR 9430791 [jid] OR 9431220 [jid] OR 9431380 [jid] OR 9431853 [jid] OR 9433352 [jid] OR 9433511 [jid] OR 9434923 [jid] OR 9434927 [jid] OR 9438200 [jid] OR 9440237 [jid] OR 9440304 [jid] OR 9442872 [jid] OR 9504817 [jid] OR 9505881 [jid] OR 9507374 [jid] OR 9510747 [jid] OR 9513551 [jid] OR 9606526 [jid] OR 9610837 [jid] OR 9611095 [jid] OR 9612493 [jid] OR 9612761 [jid] OR 9613603 [jid] OR 9613615 [jid] OR 9615568 [jid] OR 9616296 [jid] OR 9616368 [jid] OR 9616903 [jid] 14OR 9617139 [jid] OR 9705305 [jid] OR 9710936 [jid] OR 9712270 [jid] OR 9802542 [jid] OR 9808006 [jid] OR 9808874 [jid] OR 9812598 [jid] OR 9815819 [jid] OR 9876807 [jid] OR 9879082 [jid] OR 9879735 [jid] OR 9880197 [jid] OR 9881881 [jid] OR 9883260 [jid] OR 9883613 [jid] OR 9889313 [jid] OR 9890038 [jid] OR 9891973 [jid] |  |  |
| 15 |  | |  | Australia*[ad] OR Australasia*[ad] OR New Zealand*[ad] OR ANZ[ad] OR Western Australia*[ad] OR Northern Territory*[ad] OR Queensland*[ad] OR South Australia*[ad] OR New South Wales*[ad] OR Victoria*[ad] OR Tasmania*[ad] OR Sydney*[ad] OR Melbourne*[ad] OR Brisbane*[ad] OR Perth*[ad] OR Adelaide*[ad] OR Auckland*[ad] OR Wellington*[ad] OR Christchurch*[ad] |  |  |
| 16 |  | |  | **#13 OR #14 OR #15** |  |  |
| 17 |  | |  | **#12 AND #16** |  | 186 |
